# Standing lending facility in interbank market: Evidence from China

**DOI:** 10.1371/journal.pone.0284470

**Published:** 2023-05-26

**Authors:** Tiantao Guo, Yan Wang, Wanzhu Zhang

**Affiliations:** 1 School of Statistics, University of International Business and Economics, Beijing, China; 2 School of International Trade and Economics, University of International Business and Economics, Beijing, China; American University in Dubai, UNITED ARAB EMIRATES

## Abstract

We observe an anomaly that SLF quantity expansion is often accompanied by higher interbank market rates. With the Shibor bid panel, this paper empirically shows that SLF easing encourages bank risk-taking activity, and amplifies bank liquidity demand. The induced demand dominates the liquidity supply effect and leads to higher interbank rates. Moreover, the risk-taking behavior of state-owned banks is more sensitive to SLF than that of non-state-owned banks. These features make SLF a better expectation management tool than a price-based or quantity-based tool for interbank market liquidity management.

## Introduction

Liquidity facility tools have been widely used globally, especially since the 2008 financial crisis. In 2013, the People’s Bank of China adopted Standing Lending Facility (SLF) to provide liquidity to the interbank market, constructing the upper bound of the interest rate corridor with SLF rate. Monetary policy is usually transmitted through the banking system [1–4]. To understand the transmission mechanism of liquidity facility tools, some research examines the effect of these tools on the interbank market rate in some industrial economies. Most of these [5–7] reveal that a liquidity facility tool rate decrease lowers interbank rates during the crisis. This result is typically attributed to the *liquidity effect* of monetary policy [8–10], which indicates that the interbank liquidity supply marginally cuts the interbank rate. Pan and Liu [11] study the liquidity effect of SLF. A dummy variable is used to evaluate the effect of SLF, and they conclude that it can effectively lower the interbank rate level and its volatility. In addition to the liquidity effect, the *expectation effect* of liquidity facility tools should also be considered. Some literature [12, 13] explores how monetary policy affects market expectation and hence, the real economy. In the interbank market, liquidity facility tools enhance the “lender-of-last-resort” function of central banks, and thus affect the interbank rate through the shifts in market expectation formation. The expectation effect of monetary policy on bank portfolio behavior is often referred to as the *risk-taking channel*. A lower rate or greater outstanding quantity of liquidity facility tools releases the signal that market liquidity is relatively sufficient and that the risk on commercial credit is expected to be relatively low. Thus, a commercial loan, which usually bears a greater rate of return than interbank lending, becomes more appealing to banks, and vice versa. Empirical works on the expectation effect of liquidity facility tools in industrial economy interbank markets are divided. Some [6] find that liquidity facility tool easing can stabilize the market confidence and lower the interbank rate, while others [14] find that easing of facility tools like the term auction facility does not lead to more optimistic market expectation. The expectation effect of SLF in China remains under-researched. This paper aims to fill the gap empirically.

There are three main research objectives in this paper. First, we aim to determine whether the expectation effect of SLF is significant in the Chinese interbank market and how SLF affects the interbank quotation behavior of commercial banks via the risk-taking channel. There was consensus after the proposition of the Lucas Critique that all markets are affected by expectations. The financial market is one of the most affected of all. We aim to verify the mechanism that risk-taking behavior works as the mediation in SLF transmission through the interbank market. Secondly, we aim to compare the magnitude of the liquidity effect and the risk-taking encouragement effect of SLF on the interbank market. The liquidity effect refers to the increase in liquidity brought about by the expansion of SLF, while the risk-taking encouragement effect refers to the situation where the interbank market rate have an upward trend because of the induced liquidity demand. The relative size of these two effects directly determines the regulatory capacity and management mode of SLF on the interbank market interest rates. Finally, we attempt to examine the impact of bank financial heterogeneity on differential risk-taking behavior. In the Chinese financial system, state-owned banks and non-state-owned banks have different functions, and they normally exhibit heterogeneous responses to monetary policy. In addition, differences in the financial conditions of different banks can lead to differences in the degree of risk-taking behavior. Banks with different characteristics are also subject to different market funding constraints. Therefore, understanding the financial characteristics of banks and their risk-taking behavior in response to SLF stimulus provides important insights for policy making and bank liquidity management.

**Fig 1:**
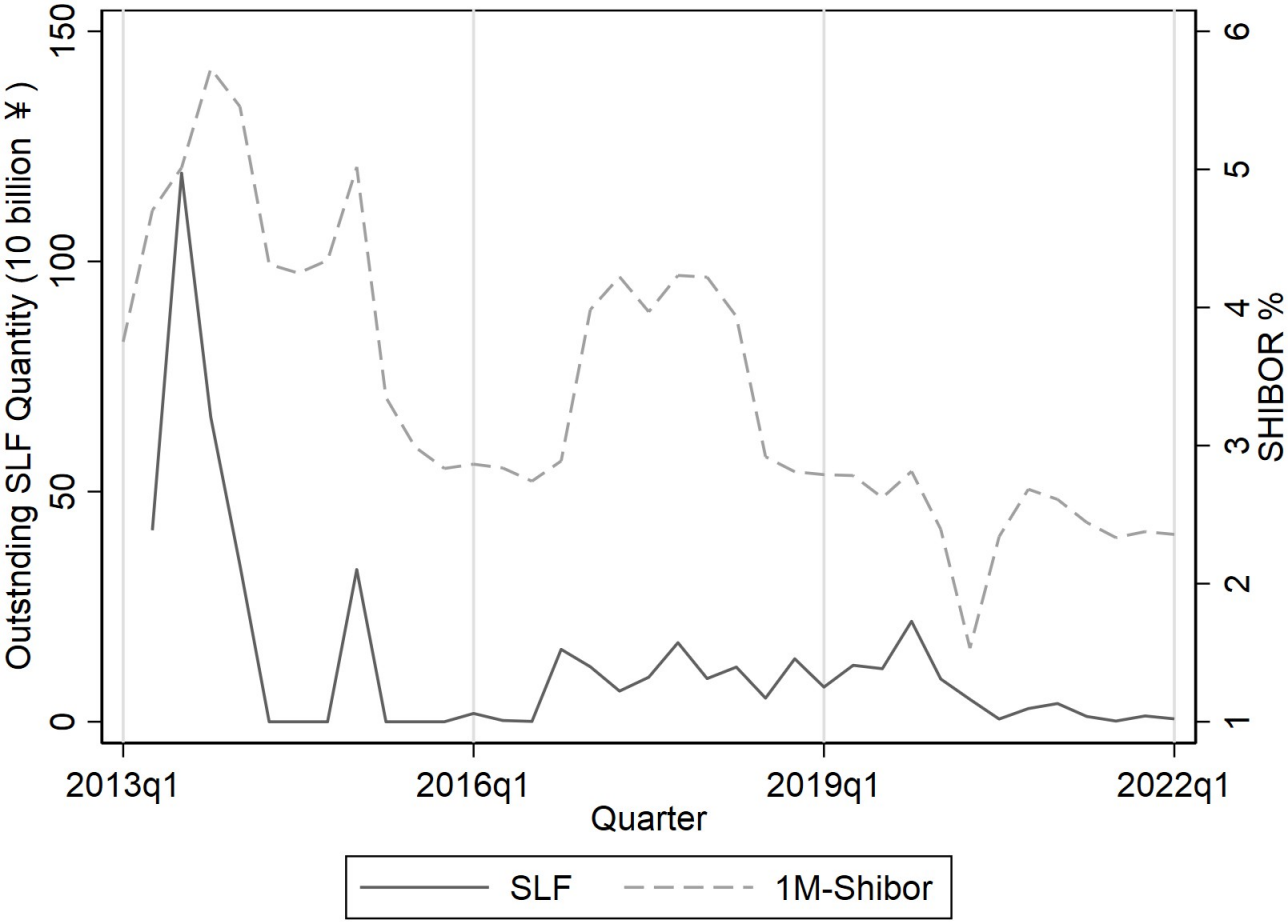
Outstanding SLF quantity and Shanghai interbank offered rate.

We observe an anomaly, as shown in Fig 1. A higher outstanding SLF quantity, which means a liquidity increase in the interbank market, is often accompanied by a higher interbank rate, and vice versa for a lower outstanding SLF quantity. Yuan et al. [15]’s study also shows this phenomenon, but they do not explain this issue in detail. Our work empirically analyzes the impact of SLF on interbank liquidity, and with both macro time-series data and panel data of interbank rate bid quotes and bank accounting indices, we attribute the anomaly to the risk-taking effect of SLF.

The risk-taking channel theory [16, 17] indicates that the monetary policy stance serves as a signal of the market liquidity risk level. Banks update their beliefs about the risk level and adjust their risk-taking behavior. They hold beliefs that the market risk level declines as outstanding SLF quantity expands (or SLF rate decreases), so they prefer lending with higher risk and return. This type of risk-taking behavior increases banks’ willingness to lend to non-bank firms and decreases their willingness to engage in interbank lending. In the short run, given constant excess reserve and deposit levels, the change in liquidity supply to a certain bank mainly consists of the change in the central bank monetary base and interbank borrowing. When the outstanding SLF quantity increases, there is a direct increase in the central bank monetary base, but there is also an indirect increase in liquidity demand and a decrease in interbank lending. When the aggregate effect of the latter influences dominates the former one, the interbank rate is hiked.

A popular empirical method to analyze the monetary policy effect is VAR/VEC models. We employ a structural vector autogression (SVAR) model to preliminarily examine the anomaly with macro time-series data, and then bank heterogeneity is taken into account with bank-level panel data. The results from both regressions align with our observation from Fig 1. Then we explore the transmission mechanism of SLF through the interbank market with panel data. Owing to the liquidity stratification phenomenon in China [18], we group our sample banks by their ownership, and we study the bank heterogeneity impact in the risk-taking process. The equity financing cost depends mostly on capital adequacy, while debt financing cost depends on asset quality. Heterogeneity in bank accounting indices and ownership influences a bank’s funding source diversity and financing cost, and thereby its risk-taking behavior.

We find that SLF, like many other liquidity facility tools, does stimulate bank risk-taking behavior in the interbank market. Our empirical results show that the bank’s risk-taking behavior is significantly positively related to the outstanding quantity of SLF in the regression. This indicates the validity of the risk-taking channel theory in the Chinese interbank market. Commercial banks tend to increase their financial risks as the market liquidity environment becomes more relaxed. Moreover, although Borio et al. [16] claim that the liquidity effect is normally greater than the effect of the risk-taking channel, our empirical analysis reveals that a higher level of bank risk-taking activity leads to higher interbank rates; that is, the risk-taking channel effect of SLF dominates the liquidity effect in the Chinese interbank market. This further indicates that SLF should be regarded more as an expectation management tool and that its stance serves as a signal of market liquidity risk. SLF is not a conventional quantity-based tool. It is not primarily used to adjust interbank market rates by changing market liquidity, as its liquidity effect is not as strong as its risk-taking effect. It is also not a conventional price-based tool. It is not primarily used to adjust interbank market rates by changing bank funding costs, as its easing has not resulted in a decrease in funding costs. Rather, it should be seen as an indicator of market liquidity safety, signaling the market liquidity environment that the central bank intends to shape. Since the risk event occurred in Baoshang Bank in 2018, several other severe risk events have occurred in China. Expectation management becomes increasingly important for financial risk event prevention and remedy. Additionally, we point out that SLF policy has differential effects on state-owned and non-state-owned banks. Generally, the risk-taking behavior of both types is encouraged by SLF expansion, while state-owned ones are more sensitive. Bank financial safety, profitability, asset quality and profit structure are key points that affect a bank’s reaction to an SLF stimulus. Specifically, in the capital market, potential investors are generally most concerned about a bank’s capital adequacy ratio. However, since state-owned banks have extremely high asset safety, investors are more interested in their profitability and therefore place more emphasis on their asset growth rate. As a result, capital adequacy ratio regulation is a stronger constraint for non-state-owned banks. In the reserve market, fund lenders are most concerned about asset quality. Unlike investors in the capital market who focus more on the liability side of a bank’s balance sheet, fund lenders in the reserve market are more concerned about the asset side of the balance sheet. Finally, because China’s non-state-owned banks are more reliant on net interest income, the income effect of SLF has a more pronounced regulatory effect on them.

The rest of this paper is organized as follows. The remainder of this section reviews the related literature, section II illustrates the methodology, section III describes the data set, section IV analyzes the empirical results, and the last section provides the conclusion.

### Literature

Our work is mainly related to two strands of literature. First is the risk-taking channel of monetary policy transmission. Borio and Zhu [17], among others, articulate the connection between the interest rate level and bank risk-taking activity. This theory claims that the policy interest rate is not only the cost of refinancing for commercial banks, but it is also a signal of the market risk level. Commercial banks infer a lower market risk level from monetary easing and thus are inclined to increase leverage ratio and invest in a riskier asset portfolio. Much empirical literature [19–22] supports this theory. Some studies [23–25] investigate the risk-taking channel of monetary policy in emerging markets, but few consider this effect in the interbank market in China. Some papers discuss about bank risk-taking behavior in China, but they mainly study the connection between bank risk-taking behavior and fintech, bank efficiency, law enforcement and bank capital structure [26–28]. Moreover, even fewer consider the risk-taking channel of SLF in the interbank market. We study the interbank effect of SLF and empirically show that the risk-taking channel not only plays a role but is the dominant mechanism in the process.

In addition to the risk-taking behavior in general, we examine how and to what extent the financial heterogeneity of banks affects their risk-taking behavior. Thus another strand of related literature studies the heterogeneous reaction of commercial banks to monetary policy. Some works [29, 30] claim that monetary policy transmission is influenced by capital regulation on banks. Kashyap and Stein [31] and Bernanke and Lown [32], among others, show that when the capital requirement is binding for commercial banks and it is costly for them to issue non-reservable liabilities or equity, expansive monetary policy may not stimulate lending. This theory is called *Capital Regulation Theory*, and it implies less reaction of banks with worse accounting indices to monetary policy. Some other works [33] claim that it is more costly for banks with worse accounting indices to replenish their reserve, so these banks depend more on the liquidity supply from the central bank and tend to react greater to monetary policy. This is the *Market Friction Theory*. Finally, Van den Heuvel [34] emphasizes the *Interest Income Theory*, which is the impact that monetary policy has on traditional bank revenue, i.e., interest income. Monetary expansion tends to steepen the yield curve when the interest rate level is not close to the zero lower bound, and it increases the traditional revenue of banks (Brunnermeier and Koby [unpublished]). Hence, the interest income theory predicts marginally better liquidity conditions of banks that rely more on interest income.

## Methodology

### Hypotheses

**Fig 2:**
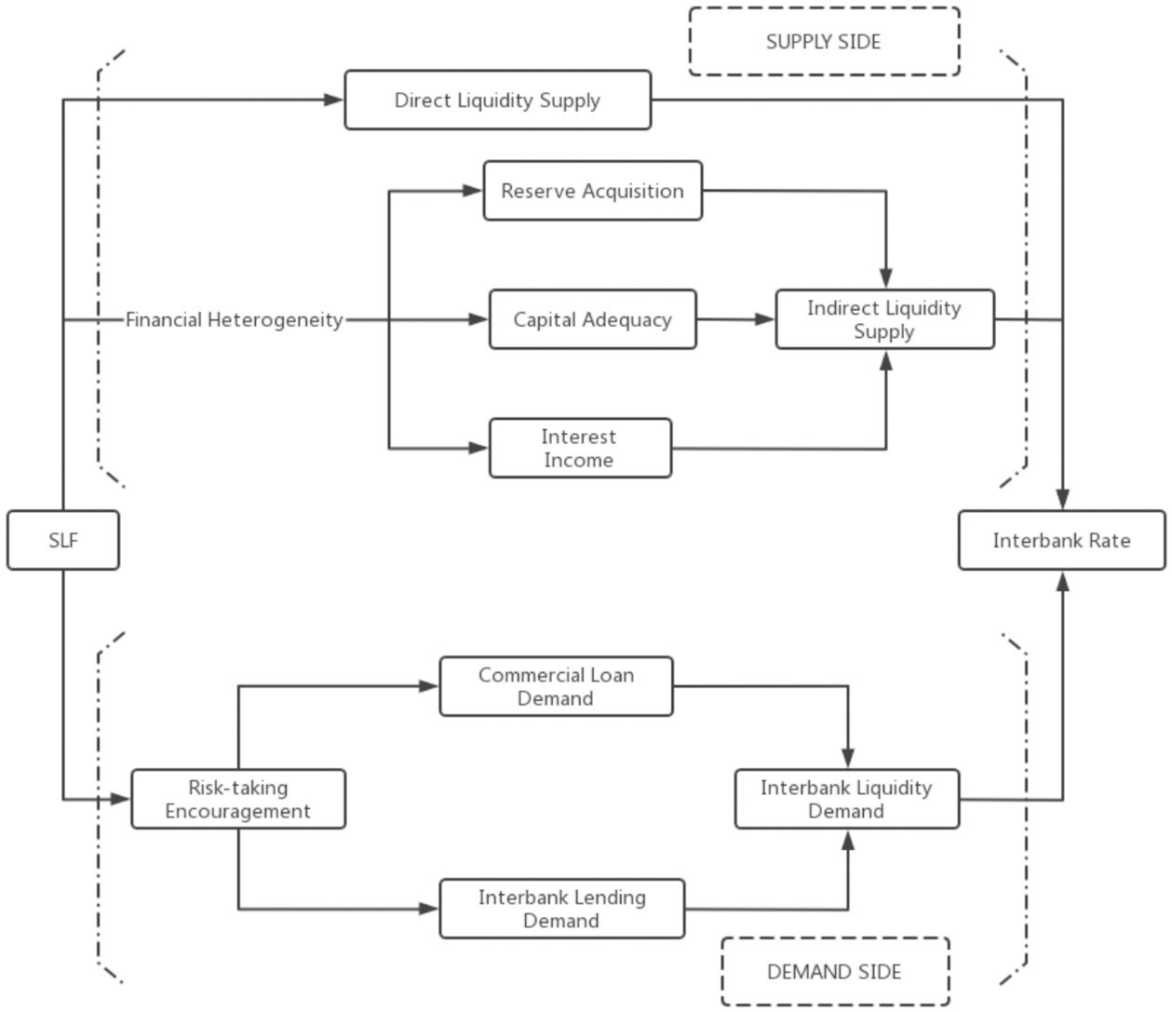
SLF transmission mechanism.

According to the risk-taking channel theory, the SLF affects both the demand and supply of reserves in the interbank market. The SLF policy stance not only implies the cost and accessibility of refinancing (liquidity supply), but it also serves as a signal released by the central bank, which affects risk preference and thereby the asset portfolio allocation of banks, and finally the liquidity demand. Further, bank accounting heterogeneity is a significant factor when we consider the reserve and capital acquisition, as well as the traditional revenue of banks. These can lead to differential reactions of banks to SLF. As summarized in Fig 2, we study both the supply and demand effect of SLF on the interbank market. For the demand side (the lower part of the graph), a higher outstanding SLF quantity encourages risk-taking behavior of banks, so their willingness corporate lending, while interbank lending decreases, which cumulatively leads to higher demand for interbank liquidity. For the supply side (the upper part of the graph), our focus is on how bank financial heterogeneity can affect SLF transmission.

To study the expectation effect of SLF, first, we argue the following:

**Hypothesis 1** *An outstanding SLF quantity expansion encourages bank risk-taking behavior, while its contraction restrains bank risk-taking behavior*.

Hypothesis (1) is a natural inference from the traditional risk-taking theory in the context of SLF. Unfortunately, it only refers to the increase in liquidity demand induced by SLF quantity expansion; it does not necessarily imply greater liquidity tension. To complete the logical chain, we need to illustrate that a riskier portfolio of banks tends to raise the interbank rate up. We hereby propose the following:

**Hypothesis 2** *A higher level of bank risk-taking activity aggravates interbank lending market liquidity tension, while a lower level of risk-taking activity alleviates the liquidity tension*.

Banks with heterogeneous characteristics face diverse liquidity situations and reveal different risk preferences. Financial indices and ownership directly affect the funding source diversity and financing costs of banks, both of which affect the substitutability of SLF. Thus, we propose the following:

**Hypothesis 3** *The financial heterogeneity of banks makes them perform differentially in the SLF risk-taking encouragement process*.

### Econometric strategies

Although some works verify the risk-taking encouragement effect of other monetary policy tools, to verify hypothesis (1), we conduct the following regression:

$$\begin{array}{c} z_{it}=\omega_{i}+\theta_{t}+\delta slf_{t-1}+\sum_{m}\lambda_{m}MP_{mt-1}+\sum_{n}\xi_{n}X_{nit}+\varphi \Gamma_{t}+\varepsilon_{it} \end{array}$$ (1)

where *z*_*it*_ is the z-score of bank *i* at period *t*, the proxy of bank risk measurement, *ω*_*i*_ is the bank individual fixed-effect term, and *θ*_*t*_ is the annual time fixed effect. *slf*_*t*−1_ is the lagged outstanding SLF quantity, and *MP*_*mt*−1_ denotes the lagged measurement of the other two monetary policy tools, OMO (m = 1) and Reserve (m = 2). *X*_*nit*_ represents accounting indices of banks, including tier 1 ratio (n = 1), net interest rate income ratio (n = 2), non-performing loans ratio (n = 3), operating income growth rate (n = 4), and asset growth rate (n = 5). The macro control variable Γ_*t*_ is the real GDP growth rate. *ε*_*it*_ is the error term. The significance of *δ* determines whether SLF has significant impact on bank risk-taking behavior, and the sign of *δ* determines the direction of the impact.

To verify hypothesis (2), we adopt the following regression equation:

$$\begin{array}{c} r_{it}=\omega_{i}+\theta_{t}+\alpha r_{it-1}+\zeta z_{it-1}+\delta slf_{t-1}+\sum_{m}\lambda_{m}MP_{mt-1}+\sum_{n}\xi_{n}X_{nit}+\varphi \Gamma_{t}+\varepsilon_{it} \end{array}$$ (2)

where *r*_*it*−1_ is the lagged term of the dependent variable *r*_*it*_ (the interbank lending market liquidity tension measured by the spread between the 1M Shanghai interbank offered rate, or Shibor, and interest rate on excess reserves, or IOER). The significance of parameter *ζ* determines whether risk-taking activity has an impact on the interbank bid rate, and the sign of *ζ* reveals the direction of the impact.

When we only examine the liquidity demand and supply of commercial banks, the greater risk appetite of banks increases the liquidity demand and reduces liquidity supply in the interbank market. Hence, combining hypothesis (1) and (2) explains the observed anomaly.

Hypothesis (3) indicates that bank heterogeneity influences its reaction to SLF risk-taking encouragement, while hypothesis (2) shows that the risk-taking behavior influences a bank’s interbank bid rate. Consequently, we can infer that bank financial heterogeneity affects a bank’s bid quoting behavior in the interbank lending market. This inference can be examined further with regression:

$$\begin{array}{cc} r_{it} & =\Xi_{it}+\delta slf_{t-1}+\sum_{n}\rho_{n}X_{nit}\cdot slf_{t-1}+\sum_{n}\sum_{m}\rho_{n,m}X_{nit}\cdot MP_{mt-1}\\ & +\tau slf_{t-1}\cdot niir_{it}\cdot cap_{it}+\sum_{m}\tau_{m}MP_{mt-1}\cdot niir_{it}\cdot cap_{it}+\varepsilon_{it} \end{array}$$ (3)

where $\Xi_{it}=\omega_{i}+\theta_{t}+\alpha r_{it-1}+\sum_{m}\lambda_{m}MP_{mt-1}+\sum_{n}\xi_{n}X_{nit}+\varphi \Gamma_{t}$. We include the sum of the interaction terms of accounting indices and all of the monetary policy tools, as well as the sum of triple interaction terms of *MP* ⋅ *niir* ⋅ *cap*. The changes of bank capital position impact the bank interest income and therefore influence the bid quotations. The triple interaction term controls for this indirect effect. According to the capital regulation theory (CRT), market friction theory (MFT), and interest income theory (IIT), which are mentioned in the literature, the expected signs for SLF interaction term parameters are listed in Table 1.

**Table 1:** Bank heterogenous reaction to SLF.

Interaction Terms	CRT	MFT	IIT
SLF×			
Cap	-	+	
NPL	+	-	
OI	-	+	
Asset	-	+	
NIIR			-

As for the estimation strategy, normally, several-term-lagged variables are employed as instrument variables to estimate dynamic panel models with the GMM. Nevertheless, a long panel data set is used in this paper, and according to Arellano and Bover [35], IV is not necessary. Moreover, to avoid too much loss of the degree of freedom, we neglect the LSDV estimators, instead, Eqs (3) and (4) are estimated with FE estimators.

### Endogeneity

Regarding econometric strategies, we should consider the possible endogeneity problem in our regressions. In all of the above regression equations, monetary policy variables are in a one-period lagged form, so the possible two-way causality between the interbank market rate/bank risk-taking behavior and monetary policy does not concern us. In addition, the interest rates on repos with rate securities as pledges for deposit-taking institutions (the *DR* rates), instead of Shibor, are considered as the potential market benchmark interest rates by the People’s Bank of China (*China Monetary Policy Report, Quarter Three, 2016*). Thus, Shibor does not directly affect the SLF policy decisions. Therefore, we do not anticipate the endogeneity problem in our regressions.

## Data

### Data sources

We employ data from multiple sources. For time-series data, Shibor is from the official data set of the Shanghai Interbank Offered Rate (The URL address of Shibor home website is <http://www.shibor.org/shibor/web/html/index.html>.). while monetary policy stance variables and real GDP growth rate are from the WIND database. For panel data, the Shibor bid quotes of banks are from the same data set as the Shibor time series above, while the bank accounting indices are from the WIND database and originally from the consolidated financial statements of all of the listed quotation banks. Moreover, 15 out of 18 quotation banks are included in our sample set because HSBC (China), China Guangdong Bank, and China Development Bank are not listed either in the Shanghai or Shenzhen Stock Exchange, and they are thus excluded. For China Minsheng Bank, we use the bid rate from Q3 2017 to Q1 2022 because it was not a quotation bank until Q3 2017. For the full list of quotation banks, see S1 Table in the Supporting information. All of the data are quarterly. The duration is from Q1 2013 to Q1 2022, 37 quarters in total (19 quarters for China Minsheng Bank). For basic statistics of macro variables, see S2 Table.

In the sample set, banks are divided into two categories, state-owned commercial banks (SOCBs) and non-state-owned commercial banks (NSOCBs). The Industrial and Commercial Bank of China, Agricultural Bank of China, Bank of China, China Construction Bank, and Bank of Communication are SOCBs, while the others are NSOCBs. For the statistics of NSOCBs see S3 Table; for those of SOCBs, see S4 Table.

Now, we focus on the time-series graph of the liquidity tension of the interbank lending market measured by 1-month-Shibor-IOER spread. (The interbank-risk-free-rate spread is a common measurement of interbank liquidity conditions, such as Christensen et al. [7], Angelini et al. [36], and Albertazzi et al. [37]). See S1 Fig. For the graph of outstanding quantity of SLF during the same period, see S2 Fig. For the bid rate curves of all of the banks, see S3 Fig, they share a similar pattern.

### Variables

Three types of variables are included in our models.

- Dependent variables. For the SVAR model, we use *liq*_*t*_, the quarterly-averaged 1-month Shibor net interest rate on excess reserve (IOER), to measure the interbank market liquidity tension. For the panel data model, we use *r*_*it*_, the spread between quarterly-averaged 1-month Shibor bids and IOER. To verify the different risk-taking behavior of SOCB and NSOCB, we employ z-score as the proxy of risk preference. The z-score, which is used in many studies [38, 39] as a bank risk index, is defined as *z* ≡ *σ*_*ROA*_/(*ROA* + *CA*), where *σ*_*ROA*_ is the standard deviation of ROA, and CA is the capital adequacy ratio. (Note that the capital adequacy ratio (CA) is different from the tier 1 ratio). Although the z-score actually measures the de facto risk level of a bank, it reveals the risk preference of the bank.
- Explanatory variables. (a) Accounting index. Our models include the tier 1 ratio (Cap), net interest income ratio (NIIR), non-performing loans ratio (NPL), growth rate of operating income (OI), and asset growth rate (Asset). (b) Monetary policy stance. Besides our analysis object SLF, two more monetary policy tools are included to serve as control variables: the open market operation (OMO) representing traditional tools and the required deposit reserve ratio of financial institutions (Reserve) representing structural tools. SLF is captured with the outstanding quantity. OMO is measured by the net open market liquidity supply.
- Macro control variable. For the panel data model, we use the GDP growth rate (GDP) to control for the market liquidity condition changes.

All of the interest rates, growth rates, and ratios are in percentage, and the units of the outstanding SLF and OMO quantity are 10 trillion CNY.

## Empirical results

### The anomaly

Before the panel data regression, to preliminarily analyze the SLF effect on the interbank rate, a structural vector autoregression (SVAR) model is employed. All of the macro variables in the regression are stationary time series according to the ADF unit root test (see S5 Table). A VAR system of the SLF and the interbank liquidity tension (*liq*_*t*_) is first established, the optimal lagged rank (see S6 Table) of the VAR is 1, and the VAR system is stationary (for the unit circle graph, see S4 Fig, for the eigenvalue table, see S7 Table). The LM test in S8 Table shows that we cannot reject the null hypothesis that there is no serial correlation, and the Jarque-Bera tests in S9 Table show that we cannot reject that the residual series of the *liq*_*t*_ equation is normally distributed, so residual series of the *liq*_*t*_ equation in the VAR is Gaussian white noise.

The SVAR writes

$$\begin{array}{c} AY_{t}=P_{1}Y_{t-1}+P_{2}X_{t}+e_{t} \end{array}$$

where vector *Y*_*t*_ = [*slf*_*t*_, *liq*_*t*_]′, *A* is the short-run restriction matrix. There are *k* = 2 endogenous variables in the model. We need the *k* × (*k* − 1)/2 = 1 restriction to identify the model. Because Shibor is not market benchmark rate in China, we suppose that in the short run, Shibor has no impact on SLF, while SLF has an impact on Shibor, which indicates that the A matrix is written as

$$\begin{array}{c} A=[\begin{array}{cc} 1 & 0\\ a & 1 \end{array}] \end{array}$$

where element *a* is a parameter to be estimated. The Granger causality test (see S10 Table) supports our restriction setting. Vector *X*_*t*_ = [*gdp*_*t*_, *omo*_*t*_, *reserve*_*t*_]′ is the exogenous variable vector. *P*_1_ and *P*_2_ are parameter matrices. Vector **e**_*t*_ denotes the error term. We believe that it is reasonable here to suppose that different monetary policy tools are used independently; moreover, the time span of our study is much shorter than the policy time lag, so monetary policy tools other than SLF and economic growth work as exogenous control variables.

**Fig 3:**
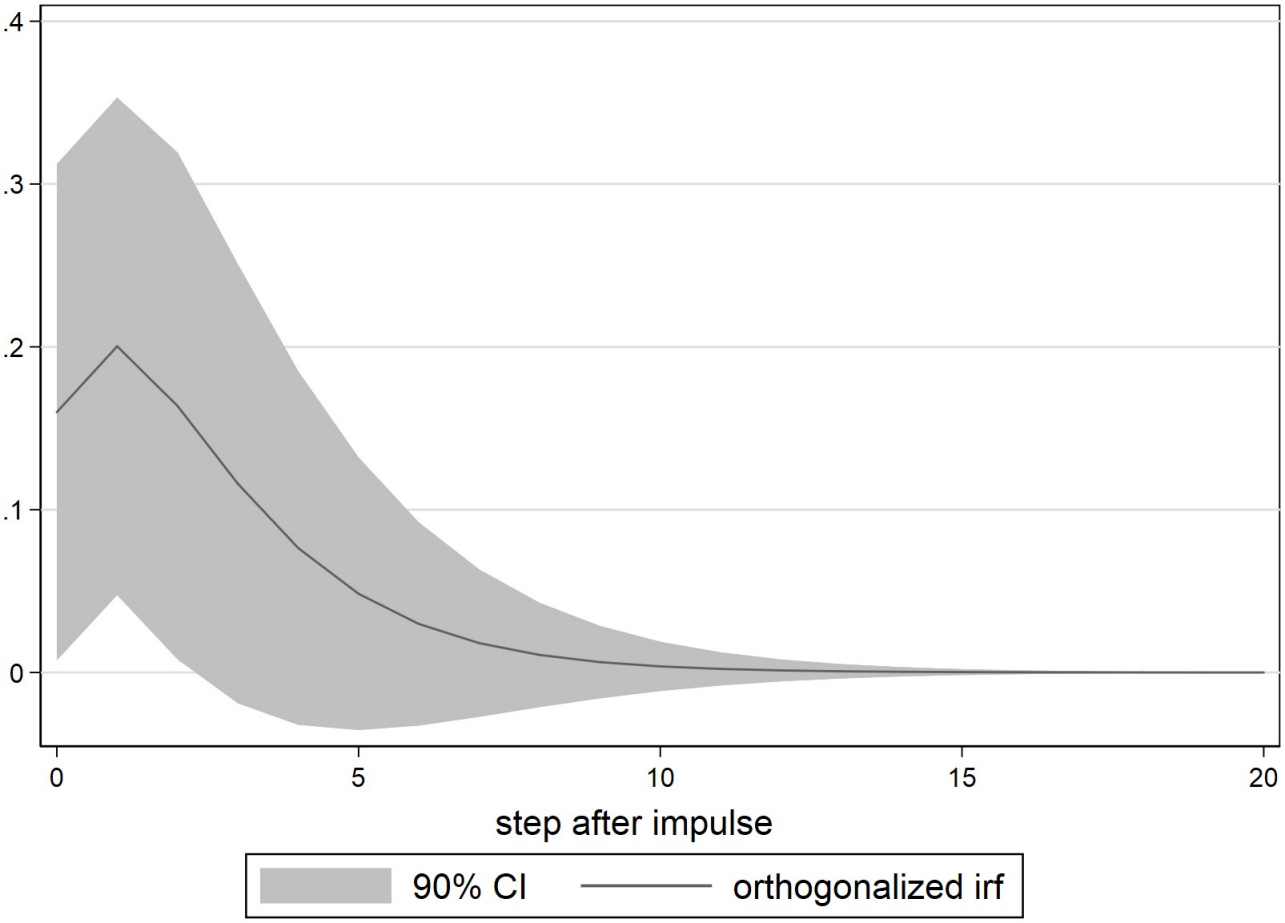
Interbank rate impulse response to SLF.

The orthogonalized impulse response function of the interbank rate to the impact of SLF expansion is plotted as Fig 3, and the 90% confidence interval is shown by the shaded area. We can observe that the instant impact of SLF on the interbank rate is raising the interbank rate. The result confirms the existence of the anomaly formally; the expansion of SLF quantity raises the interbank rate, not cuts it.

### Transmission channel analysis

To explain the anomaly, we take two steps. The first is to verify the risk-taking channel of SLF with Eq (1). The result in S11 Table is summarized in Table 2, where *insig*. means an insignificant parameter.

**Table 2:** Monetary policy effect on risk-taking.

MP tool	NSOCB	SOCB	Overall
slf	insig.	+	+

From the *Overall* column, we can observe that SLF quantity expansion spurs bank risk-taking activity generally, which verifies hypothesis (1). Specifically, every 10 trillion CNY increase of the outstanding SLF quantity adds 0.001 points to the bank z-score. This result aligns with those of some other studies, indicating the effectiveness of bank risk-taking channel. For example, with bank lending standards data in the Euro zone and the US [19], shows that a lower short-term policy rate tends to soften bank lending standards and stimulate the expansion of financial institution balance sheet. This effect is amplified when there is a high level of financial innovations. Their study shows that an increase in the overnight rate significantly soften banks standards for corporate loans, mortgage loans, and consumer loans. Similarly [22], examines the connection between monetary policy and corporate lending standards in the US with data from Senior Loan Officers Opinion Survey, and it verifies the greater appetite of banks in periods of an easy monetary policy. With credit register data in Spain [21], arrives at a similar conclusion: The relatively low short-term rate prior to the loan tends to encourage banks to lend to borrowers with bad or no credit history. These papers detail the risk-taking effects in some industrial economies. For the developing country case [20], studies the bank risk-taking behavior in the credit market in a dollarized country, Bolivia. Their study shows that a reduction of 100 basis points in the funds rate increases the likelihood of a borrower with non-performing loans getting approved for a loan by 1.1 percentage points. As for China [40], uses the non-performing ratio as the proxy of bank risk-taking behavior, and M2 as the proxy of the monetary policy stance. The regression result indicates the effectiveness of the bank risk-taking channel of money stock (M2). These studies provide strong empirical evidence for the theory of risk-taking. However, the risk-taking effect on the Chinese interbank market is under-researched. Our work fills this gap. And our regression results are consistent with the literature, namely, that monetary policy (in this case, SLF) has an encouraging effect on banks’ risk-taking. In other words, loose SLF policy may lead to an increase in money demand, which in turn may result in liquidity tension.

The columns *NSOCB* and *SOCB* present estimations with NSOCB and SOCB data, respectively. These columns indicate the differential reactions of NSOCB and SOCB to SLF. The parameter for NSOCB is insignificant, and that for SOCB is significantly positive. The regressions above mean that the risk-taking encouragement of SLF is greater for SOCBs. As for the differential impact on heterogeneous banks, the following heterogeneity analysis section examines that in depth.

Regression (1) has verified the existence of the bank risk-taking channel in the Chinese interbank market, but this does not necessarily mean that the risk-taking effect will dominate the liquidity effect of SLF expansion; in other words, the interbank market rate does not necessarily increase. So secondly, we need to verify hypothesis (2) by the regression Eq (2). Similarly to regression (1), regression (2) is conducted with SOCB data, NSOCB data, and all of the data. The result (see S12 Table) is summarized in Table 3.

**Table 3:** Mediation effect of risk-taking.

	NSOCB	SOCB	Overall
z-score	+	+	+

Parameters of the z-score for all three regressions are significantly positive. This result strongly supports hypothesis (2). The risk-taking activity of banks raises the interbank bid rate. Specifically, every point increase in the z-score adds an average of 448.253 percentage points to the bank interbank market bids. In other words, every 10 trillion CNY increase in the outstanding SLF quantity adds about 0.45 percentage points on average to interbank market bids. According to the risk-taking channel theory, we can explain the basic mechanism of the whole process. There is a substitution of interbank lending and commercial loan. Owing to the arbitrage-free principle, the expected yield for interbank lending and commercial loans should be equal. The interest rate for commercial lending is determined by the marginal productivity of capital, which is not determined by SLF in the short run. The probability of default for corporate loans is normally higher than that for interbank lending. Additionally, it is reasonable to assume that the expansion of SLF has a more significant effect on reducing the probability of default for corporate loans than on reducing the probability of default for interbank lending. Thus, the interbank lending rate has an upward trend. Meanwhile, the liquidity effect causes the interbank rate to exhibit a downward trend. Our regression unveils that the upward trend beats the downward one, and interbank rate increases.

Most literature on bank risk-taking channel refers to the above study on the effect of monetary policy on lending standards, credit availability, or credit cost. Some others study on the connection between bank characteristics [41], bank competition [42], corporate governance [43], and their risk-taking behavior. Very few compare the relative importance of the risk-taking and liquidity effects of monetary policy on the interbank market. [16] argues that liquidity effect dominates the risk-taking effect. However, the results in regression (2) reveal that in the Chinese interbank market, the risk-taking encouragement effect is greater than the liquidity effect of SLF.

Our transmission mechanism analysis actually combines the literature on the macro effects of monetary policy and the heterogeneous risk-taking behavior of banks. Literature examining the relationship between monetary policy and macroeconomic variables often uses time series data, which contain less information than panel data. On the other hand, literature studying bank risk-taking behavior tends to use panel data, but few have examined the macro effects of monetary policy. This subsection combines the two strands of literature, and emphasizes the importance of SLF as a tool for expectation management and forward guidance, not simply as a tool for liquidity management.

### Heterogeneous analysis

From the regressions above, we notice that though risk-taking channel theory generally works, so the ownership factor strengthens or weakens the risk-taking effect of SLF. In this part, we consider more observable bank heterogeneity factors, and explore how they affect bank risk-taking behaviors. By regression (1), we can verify the inference of hypothesis (3). We find that some certain interaction term parameters are significant, which supports the inference and hence hypothesis (3).

Now, we focus on the following question: How do any of the three heterogenous transmission theories mentioned above (CRT, MFT and IIT) work? The parameters of SLF interaction terms (see Table 4) are summarized in S13 Table.

**Table 4:** Bank accounting indices effect.

	NSOCB	SOCB	Overall
SLF×			
cap	-	insig.	-
npl	-	-	-
oi	insig.	+	insig.
asset	insig.	-	insig.
niir	-	insig.	-

According to Table 1, we can tell the effectiveness of CRT, MFT, and IIT for NSOCB and NSOCB, and we observe the accounting indices through which these theories affect the interbank rate. The result is presented in Table 5.

**Table 5:** Bank heterogeneity.

	CRT	MFT	IIT
Cap	⋄ ∘		
NPL		⋄ ∘ •	
OI		•	
Asset	•		
NIIR			⋄ ∘

Note: ⋄ Overall ∘ NSOCB • SOCB

CRT and MFT are supported by all of the sample sets, while IIT is not supported by the NOSB data. The effectiveness of CRT means that banks with better accounting indices are less costly in equity capital financing. Thus, they can lower their interbank liquidity bids more flexibly as SLF quantity expands. By contrast, the reaction of the relatively worse-capitalized banks is less active. Both the overall and NSOCB sample sets show that the main accounting index that transmits CRT is capital adequacy. This implies that capital adequacy regulation can help suppress the risk-taking impulse of worse-capitalized banks and reduce liquidity risk. For SOCBs only, CRT works via the asset growth rate. This means that although potential shareholders of banks generally care more about the owner’s equity (capital) when they are making their investment decisions, those of SOCBs have more confidence in the banks’ risk management capability and thus care more about their profitability.

NPL is the main accounting index that transmits MFT for all of the sample sets. This indicates that, based on traditional MFT literature [33], we find that NPL is the determinant of the reserve financing cost. Different from most equity investors, creditors (including other banks) care more about the asset side of the balance sheet. Operating income growth rate transmits MFT for only SOCBs.

IIT works for the overall sample set. Specifically, it works better for NSOCBs. NSOCBs in China are more reliant on the net interest income [44], so NSOCBs as a group tend to be affected by the net interest income to a larger extent.

The heterogeneity analysis is a beneficial supplement to the transmission channel analysis. We find that CRT, MFT, and IIT theories [29, 33, 34] operate through different factors. SOCB and NSOCB face differential challenges in the capital market and reserve market. A proper SLF policy design should consider market situations. Additionally, this result can be useful for bank liquidity management.

### Robustness

We mainly perform two robustness tests. First we consider bank heterogeneity using a dynamic panel data model:

$$\begin{array}{c} r_{it}=\omega_{i}+\theta_{t}+\alpha r_{it-1}+\delta slf_{t-1}+\sum_{m}\lambda_{m}MP_{mt-1}+\sum_{n}\xi_{n}X_{nit}+\varphi \Gamma_{t}+\varepsilon_{it} \end{array}$$ (4)

The significance of parameter *δ* determines whether SLF quantity expansion impacts the interbank market rate, and the sign determines the direction of the impact.

Considering the liquidity stratification of the banking system, regression (4) is conducted with not only the whole data set but also the NSOCB and SOCB data. The result (see S14 Table) is summarized in Table 6.

**Table 6:** SLF effect with panel data.

MP tool	NSOCB	SOCB	Overall
slf	+	+	+

The result of regression (4) agrees with that of the SVAR model above. The expansion of the outstanding SLF quantity drives up the interbank market rate. In addition, it shows that interbank bid quotes of both SOCB and NSOCB are positively driven by the outstanding SLF quantity. Specifically, according to the regression result, for each trillion CNY increase of the outstanding SLF quantity, the NSOCB interbank lending bid will increase by 1.03 percentage points, and the SOCB interbank lending bid will increases by about 1.01 percentage points. As we have analyzed in the heterogeneity part, the inclusion of bank heterogeneity does not change the main conclusion of our observation; it only adjusts the extent to which banks are affected by SLF policy. This result shows the robustness of the risk-taking channel theory in terms of bank heterogeneity.

Second, we substitute the 1-month Shibor (*r*_*it*_ and *r*_*it*−1_) in Eq (4) with overnight, 1-week, 2-week, and 3-month Shibor respectively. Some papers show the different risk-taking effects of monetary policy tools. For instance, [19] finds that the short-term policy rate has a greater effect than the long-term rate. However, few works examine the different risk-taking effects of monetary policy on the interbank market rate with different maturities. This is what we do in the second robustness test. The result (see S15 Table) is summarized in Table 7. The regression results obtained using Shibor data with different maturities are all consistent with the theory of bank risk-taking channels. Specifically, SLF policy adjustment has a greater effect on Shibor with longer maturity. Interbank rates with a longer maturity are normally believed to have greater risks, so the amplification of the expectation effect is greater. This result shows the robustness of the risk-taking channel theory in terms of maturity.

**Table 7:** Shibor with different maturities.

	Overnight	1 week	2 week	3 month
slf	+	+	+	+

Conclusively, our analysis on the risk-taking channel of SLF is robust in terms of both bank heterogeneity and interbank rate maturity. This indicates that the results of this paper can be used to analyze the SLF influence on banks with different financial features. Furthermore, the conclusions can be extended to interbank markets with different maturities.

## Conclusive remarks

This paper aims to study on the effect of SLF on the interbank lending market rates. We use an SVAR with time-series macro data to test the anomaly whereby a larger outstanding SLF quantity is often accompanied by higher interbank rates. We find that SLF quantity expansion pushes up interbank market rates. The risk-taking channel theory argues that monetary policy easing serves as a signal of lower market liquidity risk and encourages banks to make their portfolio riskier. We first verify that the risk-taking channel theory works for SLF. Then, we verify that a higher level of risk-taking activity tends to lead to higher interbank rates. Combining these two hypotheses together, we provide an explanation for the counter-intuitive phenomenon. SLF quantity expansion, as a market signal, encourages banks to bear higher risk for higher return, and this pushes the interbank liquidity demand up and pushes the supply down. Therefore, the interbank rate increases. Although some works [16] claim that the liquidity effect of monetary policy is normally greater than the effect of risk-taking effect, in the case of the SLF effect on interbank rate, the risk-taking effect dominates the liquidity effect.

Due to the liquidity stratification in China, banks in our sample are divided into two categories: state-owned and non-state-owned. The two types of banks show differential risk-taking behaviors to SLF stimulus. SOCB risk-taking behavior is more sensitive to SLF stimulus. If we examine the interaction of SLF and accounting indices, we observe that capital regulation theory and market friction theory affect SLF transmission via different accounting indices. Generally, the equity financing cost depends more on the capital adequacy while debt cost financing depends more on asset quality. Furthermore, NSOCBs in China rely on interest income more than SOCBs, so the interest income theory works especially well for NSOCBs.

Conclusively, our work suggests that SLF is more of a signal tool than a price-based or a quantity-based tool. Owing to the information asymmetry in the financial market and even the fundamental uncertainty in the economy [45], banks either lack information, or are surrounded by invalid information. Signals released by the central bank are valued with great weights in bank operating decisions. Thus, the central bank needs to use some policy tools to manage the expectation of the market [46]. SLF is a good tool for that purpose.

## Limitation

The main limitation of this work is the limited time span. As SLF was first conducted in 2013, thus we can only collect data in 2013–2022. In addition, all of the quotation banks of Shibor are large commercial banks, so the interbank behavior of medium- and small banks is not reflected in this work. Moreover, the effect of the SLF on interbank interest rates may not be linear, and it may operate in a more complex functional form. For the first two limitations, we can obtain more and richer data as time goes by. As for the third one, it can be left for further research.

## Future research

Risk-taking behavior in the interbank market is still under-researched. Our work points out the impact of SLF on bank portfolio adjustment and risk-taking behavior. Then, we may naturally ask the following question: How is the impact on the interbank market transmitted to the commercial credit market? Several possibilities exist for further research. Credit accessability is highly connected to the risk perception and risk-taking of banks. Thus, how SLF policy influences credit rationing and the financing costs of non-financial firms is an invaluable research area. In addition, SLF policy influences the risk perception of banks, and bank risk-taking behavior influences credit supply in the commercial loan market. If non-financial firms have rational expectation, as economists usually assume, how will they adjust their financing, investment, and production accordingly? The answers to these questions awaits further research.

## Supporting information

S1 Fig Liquidity tension measured by 1M Shibor-IOER spread. (TIF)

S2 Fig Monetary policy stance dynamics. (TIF)

S3 Fig 1M Shibor quotes net IOER. (TIF)

S4 Fig Unit Circle for the VAR. (TIF)

S1 Table Quotation banks of Shibor. (XLSX)

S2 Table Basic statistics for macro variables. (XLSX)

S3 Table Basic statistics for NSOCB. (XLSX)

S4 Table Basic Statistics for SOCB. (XLSX)

S5 Table ADF tests for time series data. (XLSX)

S6 Table VAR selection-order criteria. (XLSX)

S7 Table Eigenvalues for the VAR. (XLSX)

S8 Table LM test for serial correlation. (XLSX)

S9 Table Jarque-Bera normality tests. (XLSX)

S10 Table Granger causality Wald test. (XLSX)

S11 Table Risk-taking effect of SLF. (XLSX)

S12 Table Risk-taking spurs an interbank rate hike. (XLSX)

S13 Table Heterogeneity regression. (XLSX)

S14 Table Robustness: Panel data. (XLSX)

S15 Table Robustness: Different maturities. (XLSX)

S1 File (DOCX)
