# Supplementary figures and images for: Standing lending facility in interbank market: Evidence from China
Source: PLoS One. 2023 May 26;18(5):e0284470. doi: 10.1371/journal.pone.0284470 (PMC10218752; doi:10.1371/journal.pone.0284470)

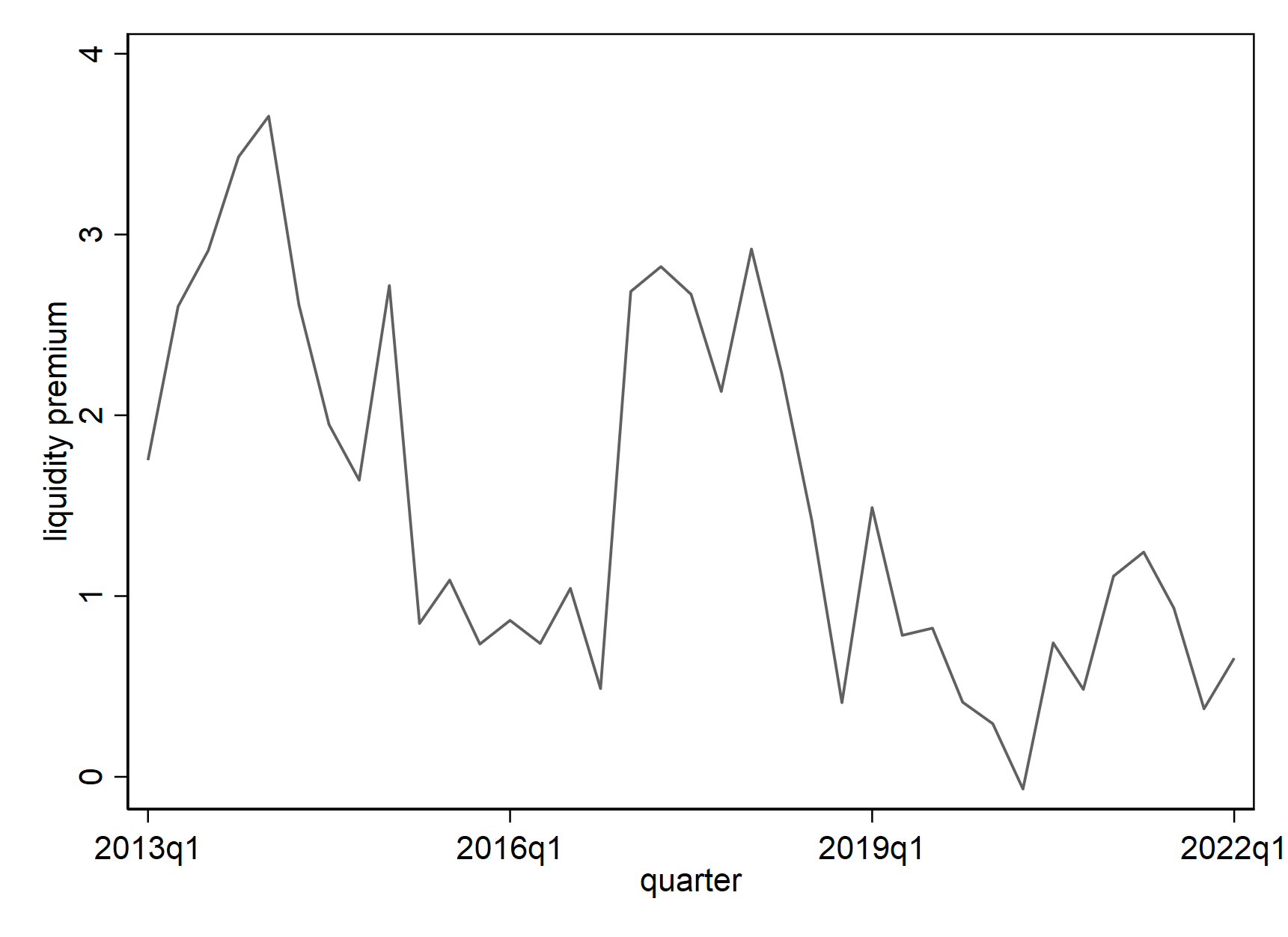

Supplement: S1 Fig — (TIF) [file pone.0284470.s001.tif]

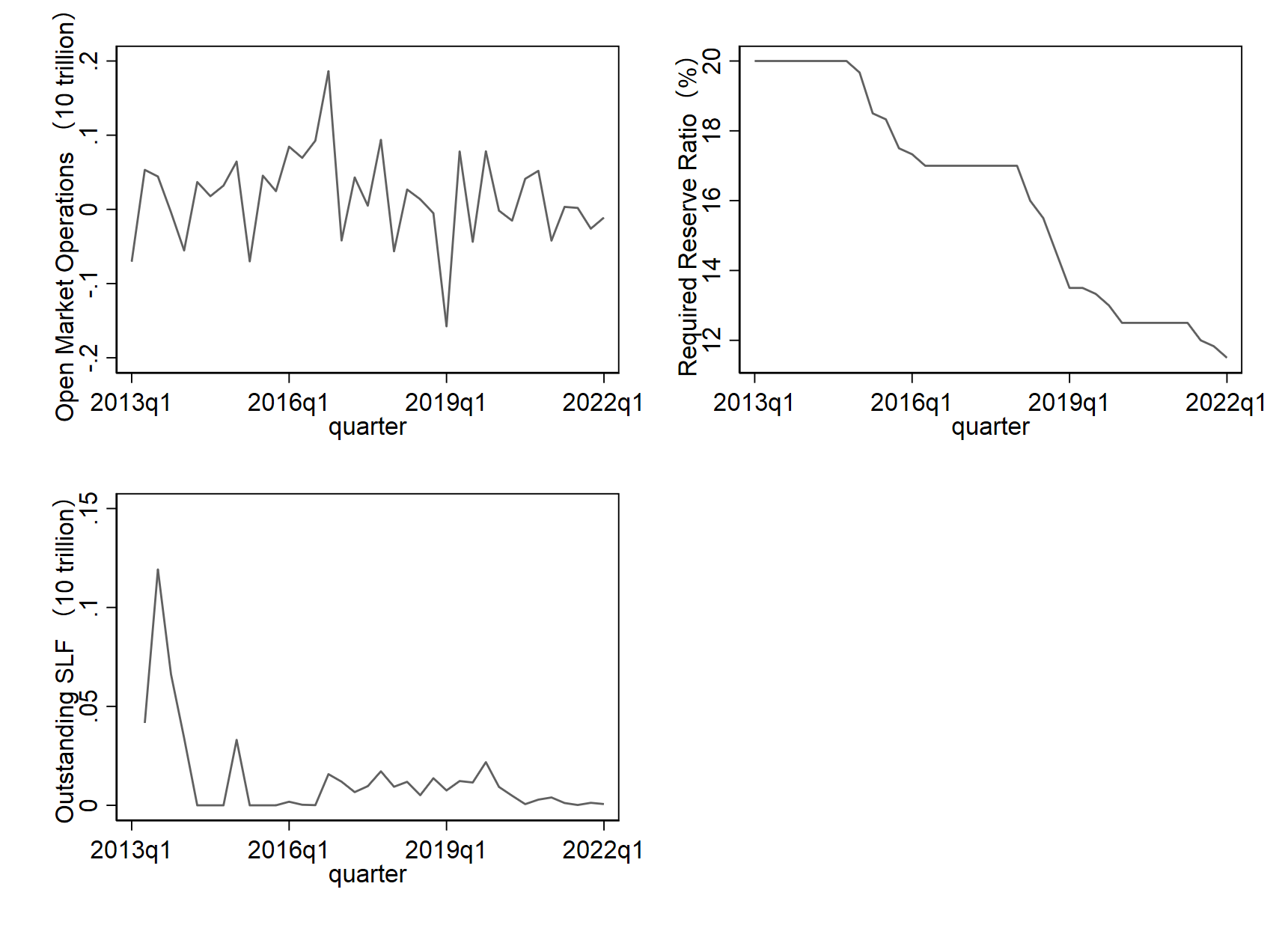

Supplement: S2 Fig — (TIF) [file pone.0284470.s002.tif]

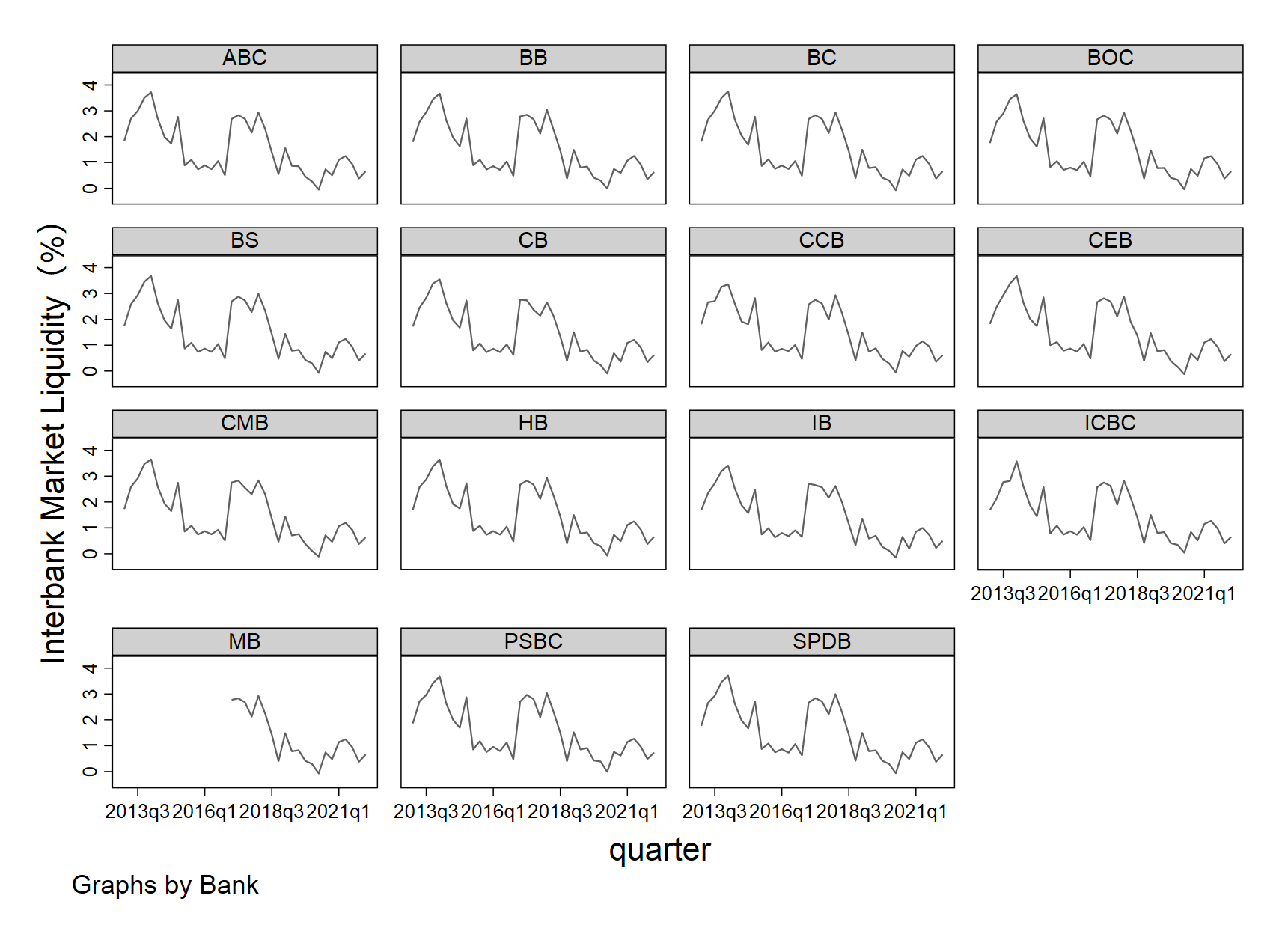

Supplement: S3 Fig — (TIF) [file pone.0284470.s003.tif]

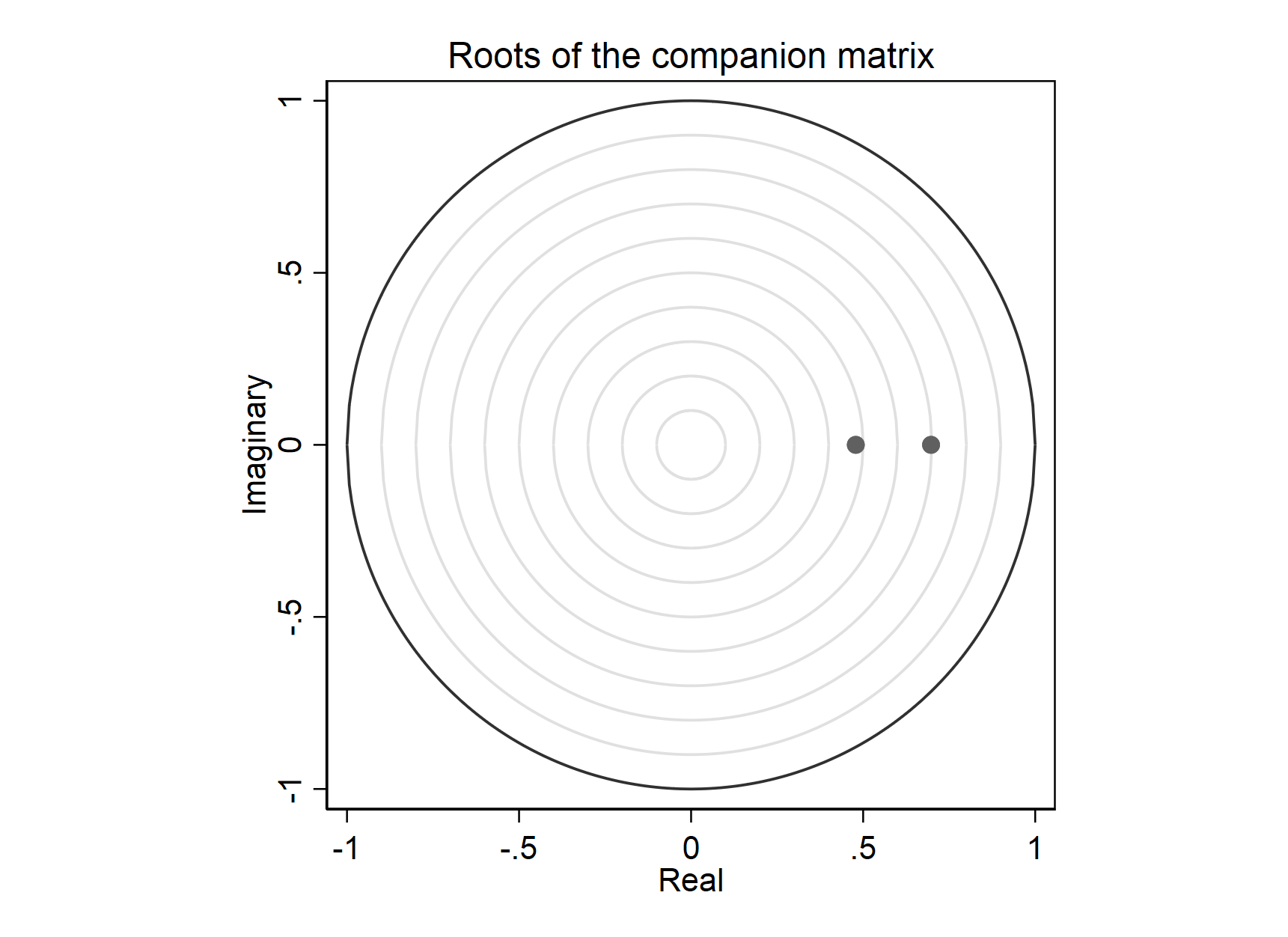

Supplement: S4 Fig — (TIF) [file pone.0284470.s004.tif]
