# Supplementary material for: Standing lending facility in interbank market: Evidence from China
Source: PLoS One. 2023 May 26;18(5):e0284470. doi: 10.1371/journal.pone.0284470 (PMC10218752; doi:10.1371/journal.pone.0284470)
Supplement: S1 File — (DOCX) [file pone.0284470.s020.docx]

1. **File List**

This zip file includes following 5 files, 2 stata code .do files, 2 data .dta files, and an instruction pdf file, namely:

- svar.do
- panel.do
- macro.dta
- panel.dta
- ReadMe.pdf

1. **Data explanation**

Table 1 explains the meaning of every variable in the data set “panel.dta”, and data set “macro.dta” is a proper subset of data set “panel.dta”. “panel.dta” is a panel data set including 15 banks and 37 quarters (2013Q1-2022Q1), “macro.dta” is a time series data set over the same time span.

| Variable | Meaning | Unit | Original Frequency |
| --- | --- | --- | --- |
| bank | English abbreviation of bank names | string | / |
| gdp | real GDP growth rate | % | quarterly |
| t | time | / | / |
| omo | open market operation quantity | ￥10 trillion | weekly |
| reserve | required deposit reserve ratio of large financial institutions | % | monthly |
| slf_ostd | outstanding quantity Standing Lending Facility | ￥10 trillion | monthly |
| shi_1M | 1-month Shibor final rate | % | daily |
| erir | excess reserve interest rate | % | quarterly |
| capital_ad | capital adequacy ratio | % | quarterly |
| tier_1 | tier 1 ratio | % | quarterly |
| niir | net interest income ratio | % | quarterly |
| npl | non-performing loans ratio | % | quarterly |
| roa | return on asset | % | quarterly |
| income | growth rate of operating income | % | quarterly |
| asset | asset growth rate | % | quarterly |
| own | ownership | dummy | / |
| sigma_roa | std. dev. of roa | / | / |
| bid_1M | 1-month Shibor bid rate | % | daily |

Table 1

1. **To replicate the analysis in the paper**

The empirical analysis is implemented with Stata 16. The SVAR model analysis is conducted by the code file “svar.do” with data set “macro.dta”, the panel data analysis is conducted by the code file “panel.do” with data set “panel.dta”.

To run the “savr.do” code with Stata 16, please first substitute the string on line 5 with the path of “svar.dta” data set. And same for “panel.do”. The results of “panel.do” would be summarized in “xls.” files named after regression equations.

***Notice***: please make sure that “outreg2” package is installed in your Stata before running code files, ***OR*** you can comment the “outreg2” commands out and choose other output commands. Alternatively, you can also comment the “outreg2” commands out and delete all “quietly” in “xtreg” commands so as to observe the results directly in Stata.

1. **Data sources**

The data sources are introduced in the paper. Original data for our variables are in different frequencies, while all data used in regressions is quarterly averaged (if the original is not quarterly).
